# *Identificazione Precoce Intervento Breve* (IPIB): the training program of the National Institute of Health--Italian National Health Service (ISS) on early identification and brief intervention on alcohol for primary health care professionals in Italy

**DOI:** 10.1186/1940-0640-8-S1-A68

**Published:** 2013-09-04

**Authors:** Emanuele Scafato, Claudia Gandin, Valentino Patussi, Tiziana Codenotti, Ilaria Londi, Silvia Ghirini, Lucia Galluzzo, Sonia Martire, Lucilla Di Pasquale

**Affiliations:** 1 National Observatory on Alcohol, National Center for Epidemiology, Surveillance and Health Promotion, National Institute of Health (NOA-CNESPS), Istituto Superiore di Sanità (ISS), Rome, Italy; 2 Careggi University Hospital, Regional Alcohol Center, Florence, Italy; 3 Eurocare Italia, Padua, Italy

## 

The Early Identification and Brief Intervention (EIBI) programme, implemented in Italy since the 1990s, is one of the most important outcomes to be achieved to reduce the level of risk linked to hazardous and harmful alcohol consumption. The NOA-CNESPS of the ISS played a pivotal role carrying out formal activities preparing a national strategy aimed at the implementation of a common standard of EIBI training now explicitly included in all national public health documents and carried out under the frame of different national/international programmes. *Identificazione Precoce Intervento Breve* (IPIB) is the national EIBI working team at the NOA-CNESPS, ISS. The team started its activities in April 2006, publishing the national strategy and organising conferences to announce, promote and disseminate the IPIB training programme. The ISS has been indicated by the National Committee on Alcohol (Consulta Nazionale Alcol) as the national provider of the training activities in tight connection with the SIA (Italian Society of Alcohology) and the Regions. IPIB training is not yet compulsory for the professionals of the National Health System, but an example of implementation at the Regional level has been the specific training experience of the Tuscany Region, a programme for all the Regions on IPIB in the workplace funded by the Centre for Control of Diseases (CCM) of the Italian Ministry of Health. The courses at the ISS, funded by the Ministry of Health and by the Presidency of the Council of Ministers, Department for Anti-drug Policies, follow a formal evaluation in terms of credits to be earned through the Continuous National Training Programme (ECM). The NOA-CNESPS, ISS, implemented 11 residential courses with the main objectives of enhancing professional skills, knowledge, attitudes and motivation of primary health care professionals when dealing with patients with HHAC. The outcome of the training courses will be presented.

